# Effect of sleep disturbances on quality of life in children with epilepsy: a cross-sectional correlational study

**DOI:** 10.1007/s11845-025-04275-x

**Published:** 2026-01-22

**Authors:** Cigdem Ceylan, Cigdem Sari Ozturk, Fatma Hanci, Aysegul Danis

**Affiliations:** 1 Health Sciences Faculty, Nursing Department, Bolu Abant Izzet Baysal University, Bolu, Türkiye; 2 Nursing Faculty, Pediatric Nursing Department, Gazi University, Ankara, Türkiye; 3 Trabzon Faculty of Medicine, Division of Child Neurology, Department of Pediatrics, University of Health Sciences, Trabzon, Türkiye; 4 Faculty of Medicine, Division of Child Neurology, Department of Pediatrics, Bolu Abant Izzet Baysal University, Bolu, Türkiye

**Keywords:** Epilepsy, Child, Sleep disturbance, Quality of life

## Abstract

**Background:**

Sleep disturbances and quality of life negatively affect each other in children with epilepsy. This study presents the relationship between sleep disturbances and quality of life in children with epilepsy in detail.

**Aims:**

The aim of the study is to investigate sleep disturbances in children aged 8-16 years with epilepsy and to determine the relationship between sleep disorders and quality of life.

**Method:**

It is a cross-sectional study. The data was collected by descriptive characteristics information form, the Sleep Disturbance Scale for Children and the Pediatric Quality of Life Inventory. 86 children with epilepsy participated in the study. Children aged 8-16 who had been diagnosed with epilepsy for at least 6 months and whose parents were literate, their parents and those who volunteered to participate in the study were included in the study.

**Results:**

54.7% of the children are female and 45.3% are male. The Sleep Disturbance scale for Children total score is 39.47 ± 9.57 (min: 28, max: 79), the Pediatric Quality of Life Inventory total score is 74.22 ± 18.98 (min: 16.30, max: 100.00). A statistically significant difference was found between seizure time, sleep disturbance and quality of life (*p* < 0.05). It was determined that there was a negative correlation between sleep disturbance and quality of life (r = -0.418, *p* < 0.05).

**Conclusion:**

As sleep disturbances increase in children, their quality of life decreases. It is important to identify sleep disturbances for improving the quality of life in children with epilepsy.

**Supplementary Information:**

The online version contains supplementary material available at 10.1007/s11845-025-04275-x.

## Introduction

Epilepsy is a noncommunicable disease of the brain characterized by seizures that cause involuntary movements that can involve partial or generalized [1] and is one of the most prevalent neurological disorders in children [2, 3]. Children with epilepsy (CWE) have seizures [4]. Epileptic seizures are usually not predictable. Epilepsy has common impact on different areas of the brain and can cause adverse health consequences for children [2, 5]. However, behavioural, emotional, social, and academic difficulties are generally higher in CWE than children with other chronic health problems [4].

Epilepsy and sleep have a complex and close relationship and influenced by various factors [6, 7]. Good sleep quality can lead to partial improvements in epilepsy, while sleep problems can negatively affect epilepsy. There are many factors for the aetiology of sleep disturbances and includes factors such as frequency of seizures, medication effects, or comorbid diseases [8, 9]. CWE experience more sleep disturbances than healthy children [10]. Sleep disturbances also lead to behaviour problems, hypersomnia, insomnia, impaired cognitive performance, lower QOL, and daytime sleepiness [4, 7, 9]. In children with epilepsy, both epilepsy and sleep problems are negatively impacting their QOL [11].

World Health Organization defines quality of life as how individuals view their life position within their society’s cultural and value systems in conjunction with their goals, expectations, concerns, and standards [12]. This essential tool plays a crucial role in gauging the effects of diseases and treatment interventions, making it suitable as the primary outcome indicator and a determinant of treatment benefits. Epilepsy is a chronic disease characterized by seizures that may take several years to manage; the quality of life can be affected by a range of epileptic and non-epileptic factors in CWE [13]. Many studies have focused on the QOL and sleep behaviours of CWE; however, there is no extensive information regarding how sleep disturbances specifically affect the QOL in CWE. Therefore, the study aimed to investigate sleep disturbances in children aged 8–16 with epilepsy and to determine the relationship between sleep disturbances and QOL.

## Materials and methods

### Study design and population

This cross-sectional study was conducted between March 23, 2022 and March 23, 2023. The data were collected from a paediatric neurology outpatient clinic in a training and research hospital in Bolu which is the city of Turkiye. The inclusion criteria of this study; (a) children aged 8–16, (b) children who had been diagnosed with epilepsy for at least six months, (c) children whose parents were literate, and (d) children whose parents and themselves volunteered for the study. The data included the descriptive characteristics information form (age, gender, duration of diagnosis, etc.), the SDSC and the PedsQL based on the parent proxy report. We reached 86 children with epilepsy.

### Measurements

In collecting the data, the descriptive characteristics information form, and the SDSC and the PedsQL were used.

The descriptive characteristics information form (age, gender, sleep duration, etc.) created by the researchers for data collection consists of 15 questions about the child’s socio-demographic characteristics [5, 8, 14–16].

Bruni et al. developed the SDSC in 1996 to assess sleep disturbances in children in the last six months. The scale consists of 26 items and six sub-dimensions and scoring is between 26 and 130. A high score on the scale indicates a sleep disorder. Ağadayı, Çelik, and Ayhan Başer (2020) were adapted to Turkish, and the value of Cronbach’s alpha was 0.79 [17].

Varni et al. developed the PedsQL in 1999 to measure the health-related QOL of children and adolescents aged 2–18. It is a general QOL scale consisting of sub-dimensions. Çakın Memik et al. (2007) were adapted to Turkish with adolescents aged 13–18. Cronbach’s alpha values were 0.82 and 0.87 for the adolescent and parent forms, respectively. Çakın Memik et al. (2008) were adapted to Turkish, with children aged 8–12. Cronbach’s alpha values were 0.86 and 0.88 for the child and parent forms, respectively. Scoring of the scale is between 0 and 100 [18, 19].

### Data analysis

The data were analysed using the SPSS 25.0 program. The research data conformity to normal distribution was tested with the Kolmogorov-Smirnov Test. Mann-Whitney U Test and Kruskal-Wallis Test were used to compare the non-normal variables. The relationships were analysed using the Spearman’s Correlation Test.

## Results

### Study participants

The characteristics of children and their families and the SDSC and the PedsQL scores were given in Table 1. The mean age of the children was 12.12 ± 2.62 years and 54.7% were female and 45.3% were male. Most of the children (40.7%) had both generalized and focal seizure type, 64.0% had seizure duration more than 10 s, 96.5% had seizure frequency less than ten times a day, and 44.2% seizure triggering factor was stress.

**Table 1:** Characteristics of children and their families and sleep disturbance scale for children and pediatric quality of life inventory scores (*n* = 86)

Variables	M ± SD		Min-Max	
Age (years)	12.12 ± 2.62		8-16	
Night Sleep Duration (hour/day)	7.94 ± 0.89		6-10	
Daytime Sleep Duration (hour/day)	0.53 ± 0.66		0-2	
Age at Diagnosis (years)	7.74 ± 3.54		1-15	
Mother’s Age (years)	40.42 ± 5.44		30-58	
Father’s Age (years)	43.24 ± 5.96		34-62	
Sleep Disturbance Scale for Children	39.47 ± 9.57		28-79	
Pediatric Quality of Life Inventory	74.22 ± 18.98		16.30-100.00	
Characteristics	n	%	SDSC	PedsQL
Age				
8-12	43	50	754.000^a^	857.500^a^
13-16	43	50	*p* = 0.140	*p* = 0.563
Gender				
Female	47	54.7	633.500^a^	672.000^a^
Male	36	45.3	*p* = 0,014	*p* = 0,034
Mother’s education status				
Primary	47	54.7	3.332^b^	9.553^b^
High school	30	34.9	*p* = 0.189	*p* = 0.008
Undergraduate and above	9	10.5		
Father’s education status				
Primary	31	36.0	0.676^b^	6.314^b^
High school	42	48.8	*p* = 0.713	*p* = 0.043
Undergraduate and above	13	15.1		
Seizure Type				
Generalized	23	26.7	1.441^b^	2.543^b^
Focal	28	32.6	*p* = 0.486	*p* = 0.280
Both generalized and focal	35	40.7		
Seizure Duration				
Less than 5 seconds	14	16.3	0.249^b^	0.972^b^
Between 5-10 seconds	17	19.8	*p* = 0.883	*p* = 0.615
More than 10 seconds	55	64.0		
Seizure Frequency				
Less than 10 times a day	83	96.5	76.500^a^	65.000^a^
10 to 20 times a day	3	3.5	*p* = 0.258	*p* = 0.161
Seizure Time				
Usually in the morning	23	26.7		
Usually at noon	4	4.7	15.423^b^	15.992^b^
Usually in the evening	13	15.1	*p* = 0.004	*p* = 0.003
Usually at night	18	20.9		
Variable	28	32.6		
Sleep Status				
Regular	67	77.9	294.500^a^	589.500^a^
Irregular	19	22.1	*p* = 0.000	*p* = 0.624
Seizure Triggering Factors				
Stress	36	44.2		
Fatigue	25	29.1	7.456^b^	5.373^b^
Insomnia	11	12.8	*p* = 0.114	*p* = 0.251
None	9	10.5		
External stimulus (light, sound etc.)	3	3.5		

^a^Mann-Whitney U, ^b^Kruskal-Wallis

The scores for SDSC ranged from 28 to 79, with a mean score of 39.47 ± 9.57. The scores for PedsQL ranged from 16.30 to 100.00, with a mean score of 74.22 ± 18.98.

### Correlation between sleep disturbance and quality of life levels

Table 2 shows the correlations between the SDSC, its sub-dimensions scores and the PedsQL, its sub-dimensions scores. Spearman’s correlation analysis revealed negative correlation between the SDSC and PedsQL (*r* = −0,418; *p* < 0.05). There was no correlation between disorders of excessive somnolence, sleep wake transition disorders, excessive sweating during sleep scores and physical health scores (*p* > 0.05), and sleep wake transition disorders, wakefulness reactions disorders scores and social functioning scores (*p* > 0.05). Negative correlation existed between the SDSC and PedsQL’s other sub-dimensions scores (*p* < 0.05).

**Table 2:** Correlation between sleep disturbance and quality of life levels

	Physical health	Psychosocial health	Emotional functioning	Social functioning	School functioning	PedsQL
Disorders of initiating and maintaining sleep	−0.235 *p* = 0.030	−0.338 *p* = 0.001	−0.378 *p* = 0.000	−0.264 *p* = 0.014	−0.299 *p* = 0.005	−0.329 *p* = 0.002
Sleep breathing disorders	−0.291 *p* = 0.007	−0.279 *p* = 0.009	−0.297 *p* = 0.005	−0.231 *p* = 0.033	−0.222 *p* = 0.040	−0.291 *p* = 0.007
Wakefulness reactions disorders	−0.284 *p* = 0.008	−0.363 *p* = 0.001	−0.345 *p* = 0.001	−0.204 *p* = 0.060	−0.403 *p* = 0.000	−0.361 *p* = 0.001
Sleep wake transition disorders	−0.208 *p* = 0.055	−0.337 *p* = 0.002	−0.318 *p* = 0.003	−0.158 *p* = 0.148	−0.369 *p* = 0.000	−0.317 *p* = 0.003
Disorders of excessive somnolence	−0.164 *p* = 0.131	−0.396 *p* = 0.000	−0.418 *p* = 0.000	−0.283 *p* = 0.008	−0.340 *p* = 0.001	−0.339 *p* = 0.001
Excessive sweating during sleep	−0.073 *p* = 0.505	−0.279 *p* = 0.009	−0.318 *p* = 0.003	−0.218 *p* = 0.044	−0.214 *p* = 0.048	−0.212 *p* = 0.050
SDSC	−0.268 *p* = 0.013	−0.447 *p* = 0.000	−0.441 *p* = 0.000	−0.283 *p* = 0.008	−0.429 *p* = 0.000	−0.418 *p* = 0.000

Spearman test

## Discussion

Epilepsy is a chronic and neurological disease. It is also characterized by seizures, affecting the paediatric population and often presents in early childhood. Sleep disturbances are common in CWE [10, 20, 21]. Difficulty falling asleep, excessive daytime sleepiness, frequent awakenings after falling asleep, sleep-disordered breathing and parasomnia are among the common sleep disturbances [7, 21]. Sleep disturbances can cause a decrease in QOL [4].

Negative correlation between sleep disturbance and QOL was found in this study. Our study’s result is similar to current studies, which found a significant negative correlation between sleep disturbances and QOL in CWE [11, 21]. In another study conducted with children diagnosed with epilepsy, the relationship between sleep behaviours and QOL was examined. Similarly, a negative relationship between sleep score and QOL score was found [22]. In our study, it is believed that sleep disturbances negatively affect QOL of CWE.

Significant negative relationships existed between the SDSC total score and the PedsQL total score, its sub-dimension scores. Similar to our study results, another current study with CWE found negative relationships between the total sleep score and the PedsQL; its sub-dimensions were psychosocial health, emotional and social functioning scores [22]. There are many sub-dimensions of QOL and different factors affecting them. One of these factors is sleep disturbances. It is believed that the results of our study showed different dimensions of QOL and the relationship between sleep disturbances in CWE.

This study found significant negative relationships between sleep breathing disorders and physical health, psychosocial health, and QOL in CWE. Disorders of initiating and maintaining sleep was also significantly negatively correlated with QOL in CWE in our study. Our study’s results are similar to current studies in CWE, which found that sleep-disordered breathing was significantly correlated with QOL, psychosocial health, and physical functioning [21], and sleep onset latency was significantly associated with QOL [23]. In our study, it is believed that sleep disturbances have significant relationships with the QOL in CWE and negatively affect the QOL of these children.

### Study limitations

This study was conducted in Central Anatolia, where there was a relationship between sleep disturbances and QOL in CWE. Therefore, it cannot be generalized. The data analysis was made from a cross-sectional study design. Thus, it can be difficult to establish temporal and causal relationships between variables.

## Conclusions

In this study, significantly negative relationship was found between sleep disturbances and QOL in CWE. It was concluded that most of them had a significant relationship when the effect of the SDSC sub-dimension scores and the PedsQL sub-dimension scores were evaluated together. It is thought that identifying sleep disorders in CWE and planning interventions to eliminate them will positively affect the QOL in these children. In addition, it is believed that this study will guide health care professionals working in neurology when evaluating QOL and sleep disorders in CWE.

## Supplementary Information

Below is the link to the electronic supplementary material.

Supplementary Material 1

Supplementary Material 2
